# Editorial: Intersectionality and Identity Development: How Do We Conceptualize and Research Identity Intersectionalities in Youth Meaningfully?

**DOI:** 10.3389/fpsyg.2021.625765

**Published:** 2021-02-02

**Authors:** Margarita Azmitia, Katherine Cumings Mansfield

**Affiliations:** ^1^Psychology Department, University of California, Santa Cruz, Santa Cruz, CA, United States; ^2^Department of Educational Leadership and Cultural Foundations, University of North Carolina at Greensboro, Greensboro, NC, United States

**Keywords:** identity intersectionalities, youth/young adults, gender/sexuality, race/ethnicity, education, globalization, transnational

The concept of *intersectionality*, an analytic framework or lens for investigating how social identities are configured by power and oppression, was introduced by Crenshaw (1991) in the *Stanford Law Review*. In this publication, Crenshaw argued that gender, race, social class, and sexuality are inseparable and non-hierarchical dimensions of oppression that are not recognized by current legal tools that focus on race or gender as separate constructs. While Crenshaw's theory-building focused on the inability of the United States Justice System to adequately address the different experiences for Black women who are survivors of domestic abuse, the concept has been adopted and adapted to critical studies beyond the field of law.

For example, a special issue of *The Journal of Negro Education* focused on the unique experiences of African American girls in American schools. For instance, Watson (2016) used an intersectional approach (hooks, 1989; Collins, 2000; Crenshaw et al., 2015; Morris, 2015) to show how Black girls' experiences in school were far different from those of white girls and Black boys. Black girls were subject to different rules in terms of dress code and demeanor, experienced school as a hostile rather than welcoming place, and recommended schools institute affinity groups to provide safe spaces for Black girls to build positive relationships and self-esteem. In another example, Jacobs (2016) used hooks (1992) concept of oppositional gaze as a frame to argue that Black girls' identity development was directly tied to media images and that developing a critical lens and analytic skills via the purposeful selection of curriculum and pedagogy were central to their positive development.

Psychology has also showed an increased interest in the construct of identity intersectionalities. For example, in a recent special issue in *Developmental Psychology*, Galliher et al. (2017) proposed an integrative model for investigating identity processes and content that is situated in historical and contemporary contexts of prejudice and oppression of minoritized youth. Across the world, activist youth and their allies remind society that Black lives matter by working for social justice and equality in a variety of domains and institutions, including immigration, prison reform, and equity in education. Educational leaders are also reducing inequalities by having difficult conversations about gender, race, sexuality, and other categories of oppression with students and staff (cf. Mansfield and Jean-Marie, 2015; Sue, 2015).

While the concept of intersectionality helps us understand the spaces that youth occupy as a function of their unique personal and social identity configurations, further research and theory building on the development of identity intersectionalities and their consequences for young individuals' daily lives is needed. It is essential that future research specifically operationalizes the intersectionality construct as it is used in empirical investigations to take advantage of the potential power of Kimberlé Crenshaw's ideas and to prevent turning her insights into an empty construct that results in generating an endless list of identities without meaningful analyses and applications (cf. Collins, 2019; Harris and Patton, 2019).

In response to these concerns, we invited researchers to submit their work on intersectionality that included a discussion of developmental theory and an overarching research agenda that addressed questions forwarded by Azmitia and Thomas (2015, citing Cole, 2009):

(1) Who is included in a social identity category? And are there diversity levels of within-group variability in a social identity, such as gender, race, class, or sexuality? When does the dissimilarity between group members render the identity category incoherent?
(2) What roles do inequality and oppression play in the construction of identity intersectionality? We invited authors to continue Crenshaw's discussion around inequality in the legal system, concentrating on how power and oppression contextualize youth's daily lives. We suggested, for example, that they problematize the construct of multiple jeopardy, i.e., that the more minority identities youth have, the more powerless they will feel in contemporary society. We expected that authors might also address whether and how these multiple minoritized identities serve as a source of pride and power for youth, particularly those engaged in social activism.
(3) Are there similarities in the intersectional experiences of youth who vary in their social identities? For example, is there a common ground for youth who experience different identity configurations and oppressions?

The five papers in this special issue present interdisciplinary, theoretical, and empirical roadmaps for applying an intersectional lens to identity development. In the first paper, Ghavami et al. compare and contrast multiple identity and intersectional theories to frame the results of two studies that explore potential relationships between perceived gender identity, ethnic/racial identities (ERI), sexual orientation, and body weight with peer affiliation and discrimination in urban middle schools. Using latent profile analysis, they show how early adolescents' perceptions of their typicality in these social categories, and how they intersect in their peer experiences, affects their experiences of discrimination and perceptions of school belonging.

In the second paper, Parra and Hastings focus on the lived experiences of sexually diverse Latinx youth, who are often invisible due to cultural and religious privileging of heterosexuality and because research on sexually diverse populations has primarily focused on white youth. Parra and Hastings show how the effects of prejudice and discrimination on well-being and health can be assessed by integrating daily surveys with biological measures of stress, i.e., salivary cortisol. In developing their identities, ethnic/racial minority youth have to negotiate their minoritized identity in the context of American identity (AI) and the status of their minoritized group at school and the larger society. Hastings and Parra conclude that heterosexism and racism intersect in the identity negotiations of sexually-diverse Latinx youth and challenge their ability to integrate these minoritized identities. Yet, doing so predicts positive social adjustment and well-being.

In the third paper, Cheong et al. continue the discussion of ERI AI identity intersectionalities to report on a longitudinal study of how diverse adolescents' growing awareness of their social status relative to other groups at school and their communities contours their intersectional construction of ERI and AI. To this end, they use a person-centered, quantitative approach to construct profiles of adolescents who strongly or weakly identify with these identities. They then link these profiles to adolescents' experiences of discrimination, mental health, and school engagement. As predicted, members of the weakly-identified profile, low in both ERI and AI, reported higher levels of discrimination and depression, perhaps due to feelings of marginalization. Unexpectedly, those in the moderate ERI and AI group had the lowest school engagement, which the authors interpret as resulting from their growing awareness of discrimination toward their ethnic/racial groups. Consistent with Tajfel and Turner, 1979 Social Identity Theory, adolescents high in ERI and moderate in AI had higher school engagement and lower perceptions of discrimination and depression, underscoring the protective role of ERI for school engagement and mental health.

Much influential work on intersectionality has been carried out outside the U.S. (c.f., Butler, 2011; Meer and Müller, 2017; Mollet, 2017; Hurtado, 2018). Moffitt et al. discuss the challenges of including race in intersectional research in Europe because the atrocities of World War II have led to a reluctance to openly discuss how racial prejudices and discrimination affect people's everyday lives and opportunities. As Europe opens its borders to immigrants from Africa, Asia, and the Middle East, intersectional research also has to confront the vestiges of colonialism that views immigrants with a deficit lens. Using Muslim youth as a case study, Moffitt et al. argue for a more dynamic operationalization of the social categories of race, religion, and immigration so developmental psychology can embark in intersectional research that strengthens theories and methods in ways that capture the lived experiences of minoritized European youth.

In the last paper, Grabe brings us back to Crenshaw's original work on intersectional experiences of women of color in the legal system by sharing her research in Nicaragua and Tanzania on how programs that confer land ownership to women increase their status in their families and communities and reduce domestic violence. In describing the similarities and differences in working with activist women and youth in Nicaragua and Tanzania, Grabe provides a road map for collaborating with communities so that investigations not only contribute to theory and research, but also improve the everyday lives of women and youth in those communities.

Taken together, these articles enrich the methodological toolkit for studying intersectionality, underscoring that quantitative, qualitative, and mixed methods designs not only can coexist, but work together toward a better understanding of identity intersectionality development, also augmenting interdisciplinary scholarship and practice. As readers engage with these authors, we recommend they foreground the questions we posed earlier to apprehend the richness and diversity of these interdisciplinary offerings. Reflecting on Grabe, as the researchers carried out their empirical work, they also developed their own intersectional identities, often collaborating with youth and communities to achieve social justice. Likewise, as researchers, we too hope to develop dynamic intersectional lenses about ourselves and our work.

## Author Contributions

All authors listed have made a substantial, direct and intellectual contribution to the work, and approved it for publication.

## Conflict of Interest

The authors declare that the research was conducted in the absence of any commercial or financial relationships that could be construed as a potential conflict of interest.
